# Chorio-retinal vessel density in women affected by functional hypothalamic amenorrhea: a monocentric observational cross-sectional study to evaluate the impact of hypoestrogenism on chorio-retinal vascularization

**DOI:** 10.1007/s00404-024-07603-1

**Published:** 2024-07-04

**Authors:** Alice Diterlizzi, Anna Tropea, Emanuela Angelini, Valentina Cestrone, Romina Fasciani, Annamaria Merola, Giovanna Notaristefano, Martina Asia Policriti, Teresa Polimeno, Monia Ranalli, Maria Cristina Savastano, Ghazal Tannous, Valeria Versace, Stanislao Rizzo, Giovanni Scambia, Antonio Lanzone, Rosanna Apa

**Affiliations:** 1 Department of Women’s and Children’s Health Sciences and Public Health, Fondazione Policlinico Universitario A. Gemelli, IRCCS, L.Go Agostino Gemelli, 8, 00168 Rome, Italy; 2 Università Cattolica del Sacro Cuore, Largo F. Vito 1, 00168 Rome, Italy; 3 Department of Ophthalmology, Fondazione Policlinico Universitario A Gemelli, IRCSS, L.Go Agostino Gemelli, 8, 00168 Rome, Italy; 4 Department of Statistical Sciences, Sapienza University of Rome, 00185 Rome, Italy; 5 National Research Council (CNR), “Istituto Di Neuroscienze”, Pisa, Italy

**Keywords:** Functional hypothalamic amenorrhea, Estrogens, Vascular dysfunction, Chorio-retinal circulation, Vessel density

## Abstract

**Purpose:**

Functional hypothalamic amenorrhea (FHA) is characterized by an estrogen deficiency which in turn can cause vascular dysfunction. The aim of this study is to evaluate any changes in the chorio-retinal circulation in patients affected by FHA. 24 patients with FHA and 24 age-matched controls underwent a gynecological evaluation and an OCT angiography (OCTA) to study chorio-retinal vascularization.

**Results:**

OCTA in FHA patients showed an increase in vessel density in the choriocapillaris (CC) layer (both in the fovea area, at 5% *p* value = 0.037 and in the whole area, at 5% *p* value = 0.028) and an increase in vascular density in the deep fovea (DVP) (at 10% *p* value = 0.096) in the whole district compared to controls. Simple linear regressions show a significant negative association between CC vessel density and insulin (*p* = 0.0002) and glucose values (*p* = 0.0335) for the fovea district and a negative association between DVP vessel density and endometrial thickness (at 10%, *p* value: 0.095) in the whole district.

**Conclusion:**

Our study shows that CC vessel density is increased in women affected by FHA. This could represent a compensation effort to supply the vascular dysfunction caused by estrogen deficiency. We also found an increasing trend in vascular density in DVP associated with the decrease of endometrial thickness, an indirect sign of estrogenization. Considering that these changes occur in absence of visual defects, they could be used as a biomarker to estimate hypoestrogenism-induced microcirculation changes before clinical appearance.

## What does this study add to the clinical work

**Table:** 

OCTA study of chorioretinal circulation in FHA patients is a reasonably promising biomarker to evaluate preclinical hypoestrogenism-induced microcirculation changes in eye.	

## Introduction

Functional hypothalamic amenorrhea (FHA) is defined as the absence or cessation of menstrual cycles due to suppression of the hypothalamic–pituitary–ovarian axis with GnRH pulsatility deficiency, without any associated organ injury. It is related to mental stress and excessive physical exercise with consequent energy deficit and/or eating disorders. This clinical condition, although potentially reversible, is characterized by estrogen deficiency which can expose over time to the risk of developing osteopenia/osteoporosis and other clinical manifestations [1].

Moreover, in cases of hypoestrogenism, the effects on the circulatory system of these hormones should be considered. It is known that estrogens play a key role in modulating the functionality of the cardiovascular system: coronary and peripheral vessels contain estrogen receptors through which they act in various mechanisms [2, 3]. Many studies demonstrate an endothelial dysfunction in case of hypoestrogenism [4, 5].

Indeed, some authors demonstrated an endothelial dysfunction with an increase in vascular resistances in patients with functional hypothalamic amenorrhea [3, 6, 7]. Also, as emerged from the Women’s Ischemia Syndrome Evaluation, in a study of coronary angiography in women with coronary risk factors, estrogen deficiency was associated with low levels of LH and FSH correlating with the presence of coronary artery disease, and the diagnosis of hypoestrogenemia of hypothalamic origin was found to be an independent risk for coronary heart disease. For this reason, FHA is considered to lead, according to some evidence, to an increase in cardiovascular risk at a young age [8].

However, the exact impact of estrogen deficiency on vascular districts is yet to be defined.

Considering the eye involvement, 17b-estradiol (E2) has been demonstrated to play a crucial function in retinal vascular abnormalities. E2 therapy boosts retinal blood flow, preserves the retinal nerve fiber layer in ovariectomized rats, and inhibits retinal glial cell swelling in rats. [9]. The consequences of estrogen deficiency on the eye have been studied by different authors, but remain unclear.

Obrubov et al. in 2013 [10] found ultrastructural changes in retinal layers in experimental hypoestrogenism in female rabbits. However in humans, Moschos et al. found differences in the thickness of macula among patients with restrictive anorexia compared to those with binging/purging type [11] and a reduction in the thickness of the choroid which has been related to peripheral endothelial dysfunction [12].

The aim of this study was to investigate if estrogen deficiency could lead to any changes in chorio-retinal circulation layers (deep and superficial vascular plexus and choriocapillaris layer, both in the fovea and in the whole area) in patients with FHA, compared to age-matched healthy controls.

The recent introduction of optical coherence tomography angiography (OCTA) allowed to investigate these circulations and to obtain a post-processing quantitative analysis of vascular parameters [13].

To the best of our knowledge, this is the first study which analyzed the intraretinal plexus and the choriocapillaris circulation with OCTA in hypothalamic amenorrhea patients.

## Methods

This was a monocentric observational cross-sectional study. The study protocol was approved by the Ethics Committee of Fondazione Policlinico Universitario A. Gemelli IRCCS (Prot N. 0011981-31/03/2021; ID3961), and all participants were enrolled only after receiving an explanation of the purpose and nature of the study. It was conducted in accordance with the Declaration of Helsinki, as revised in 2013. Informed consent was obtained from all subjects.

Twenty-five patients with FHA and 25 age-matched healthy controls were recruited from the Department of Women’s Health Sciences, of the Child and Public Health of Fondazione Policlinico Universitario A. Gemelli IRCCS.

At the time of recruitment, patients were selected according to the inclusion criteria and were subjected to a full clinical evaluation, which included routine tests to assess their demographic, clinical, instrumental, and laboratory characteristics.

Patients were selected according to the inclusion criteria: age in the range of 18–37 years, a diagnosis of FHA based on the absence of menstrual cycles for at least 6 months [14], with a negative medroxyprogesterone acetate (MAP) test and circulating values of estrogens less than 50 pg/ml, and FSH and LH less than 10 mIU/ml, after performing a GnRH test to exclude a pituitary impairment and excluding other anatomical–functional pathologies. Exclusion criteria for all participants were: oral contraceptive consumption in the previous 3 months; autoimmune diseases; coexisting polycystic ovary syndrome; diabetes mellitus; major surgery in the last 3 months; other hormonal dysfunctions (hypothalamic, pituitary, thyroidal, or adrenal causes). Ophthalmological exclusion criteria were high myopia (superior to three diopters), diabetic retinopathy, uveitis, previous ocular surgeries, and low quality index of structural OCT and OCTA scans (lower than 7/10).

All participants underwent a pelvic ultrasonography using a 6.5 MHz endovaginal probe (Samsung WS80A) and laboratory tests to assess their hormonal values. They also underwent a complete ophthalmological examination, comprehensive of OCTA (Solix full-range OCT, Optovue Inc., Freemont CA, USA). Structural OCT scans consisted of a single line passing horizontally and vertically in the fovea region. OCTA scans performed were 6.4 × 6.4 mm, centered on the fovea.

OCTA results were acquired from the Angiovue Retina software that automatically analyzes the vessel density (VD) of the superficial capillary plexus (SCP), deep capillary plexus (DCP), and the choriocapillaris (CC) layer in *whole image* and in *fovea-grid-based image* [15]. Angioanlytics algorithm (default embedded automated post-processing image acquisition) allowed to calculate the vessel density in the superficial vascular plexus (SVP), deep vascular plexus (DVP), and choriocapillaris layer (CC) in the macular region.

All the results were obtained using the R statistical software (free open source). To assess if the differences between the ophthalmological and gynecological data of cases and controls were statistically different, we conducted a two-tailed *t* test assuming equal variances in the two populations, at a significant level of 0.05.

## Results

In Table 1, we present the clinical characteristics and laboratory and instrumental findings of both FHA patients and healthy controls as the mean, standard deviation (St. dev), and interquartile range (iQR) and the statistically significant differences based on the *p* value of the two-tailed *t* test.

**Table 1:** Statistical differences between clinical and laboratory characteristics of FHA patients and healthy controls

Variables	FHA patients			Healthy controls			FHA patients vs healthy controls
	Mean	St. Dev	IQR	Mean	St. Dev	IQR	*p* value
Age (years)	24.500	6.186	9.500	25.278	3.304	1.000	ns
Duration of amenorrhea (months)	17.667	14.619	10.750	/	/	/	ns
Height (m)	1.632	0.077	0.095	1.667	0.070	0.095	ns
Weight (kg)	50.496	5.668	6.050	59.481	4.194	6.000	** < 0.0001**
BMI (kg/m^2^)	18.966	1.679	1.798	21.149	1.353	1.574	** < 0.0001**
FSH (UI/ml)	5.112	2.318	3.575	7.317	1.370	2.425	** < 0.0001**
LH (UI/ml)	2.183	2.136	4.000	5.876	1.940	3.000	** < 0.0001**
E2 (pg/ml)	30.083	12.321	14.250	50.411	14.089	17.000	**0.001**
AMH (ng/ml)	4.182	1.828	2.742	3.667	1.506	1.942	ns
CRP (mg/L)	1.732	3.501	0.000	0.817	1.060	0.000	ns
TSH (µUI/ml)	1.556	0.715	0.812	1.538	0.486	0.780	ns
FT4 (pg/ml)	10.100	1.200	1.625	11.606	1.266	2.025	ns
PRL (ng/mL)	4.942	2.634	1.850	11.356	6.859	5.625	**0.001**
Insulin (µU/mL)	3.900	2.557	2.175	5.736	2.073	3.300	**0.021**
Glucose (mg/dl)	73.043	6.725	8.500	79.154	5.505	7.000	**0.006**
Endometrial thickness (mm)	2.096	1.696	2.100	/	/	/	

*ns* not significant

Bold values indicate the statistically significant differences

Patients in the study group suffered a mean period of amenorrhea of 17.667 months. The mean weight reported in the study group was 50.496 kg and in the control group 59.481 kg (*p* < 0.0001). Cases had a mean BMI of 18.966 compared to a mean BMI of 21.149 in the controls (*p* < 0.0001).

17b-estradiol (E2) level in the cases was 30.083, while on the other hand in the eumenorrheic controls it was 55.944. Hence, E2 levels were significantly lower in the study group (*p* = 0.001).

We also underline an interesting significant difference between insulin values, reduced in FHA patients (3.900 µU/mL) vs healthy controls (5.736 µU/mL), *p* = 0.021, as well as lower levels of fasting glucose in FHA patients than in healthy controls (73.043 mg/dl vs 79.154 mg/dl, *p* = 0.006).

Finally, the levels of prolactin were significantly reduced in FHA patients compared to healthy controls (4.942 vs 11.356 ng/mL, *p* = 0.001).

The OCTA results for cases and controls are summarized in Table 2.

**Table 2:** OCTA variables in FHA patients vs healthy controls

Variable	FHA	Healthy controls	FHA vs healthy controls
	Mean	Mean	*p* value
SVP whole area	51.043	51.185	0.816
SVP fovea area	28.850	30.481	0.268
DVP whole area	56.285	54.264	**0.096**
DVP fovea area	32.164	34.108	0.278
CC whole area	69.316	67.968	**0.028**
CC fovea area	65.539	62.656	**0.037**

**Fig. 1:**
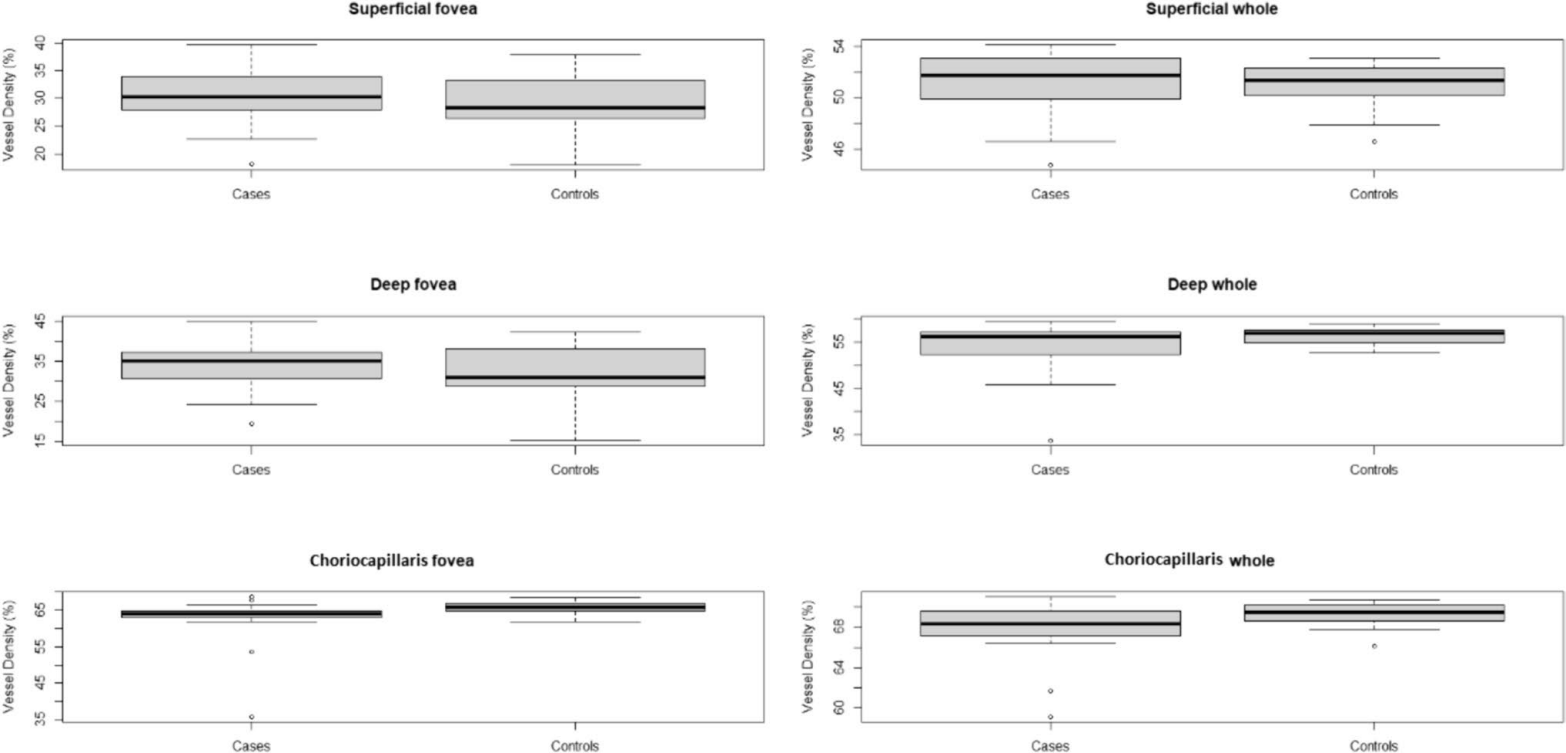
Box plots of the distributions of vessel density for superficial fovea, deep fovea, and choriocapillaris fovea in the cases and controls

The vessel density of the superficial vascular plexus in the *whole area* was 51.185 in the healthy subgroup and 51.043 in FHA patients (*p* = 0.816), while in the *fovea area* it was 30.481 in healthy controls and 28.850 in FHA patients (*p* = 0.268). Vessel density in the deep capillary plexus in the *whole area* was 54.264 and 56.285, respectively, in the healthy and FHA groups. Vessel density of the deep vascular plexus in the *fovea area* was 34.108 in controls and 32.164 in patients. A difference was found among the two groups in the deep vascular plexus *whole area* (at 10%, *p* = 0.096), while no difference was observed in the deep *fovea area* (*p* = 0.278). In the choriocapillaris layer, the vessel density in the *whole area* was 67.968 in healthy controls and 69.316 in FHA women, while in the *fovea area* it was 62.656 and 65.539, respectively, in controls and in FHA patients. A statistically significant difference was detected between the two groups for both CC *whole area* (*p* = 0.028) and *fovea area* (*p* = 0.037) (Fig. 1).

Finally, to analyze the dependence among variables, we fitted different simple linear regression models, considering as response variables (dependent variables) OCTA values for SVP, DVP, and CC layers and as a set of regressors: months of amenorrhea, E2, endometrial thickness, FSH and LH, insulin, glucose, TSH, and FT4. In the sequel, we report only the most relevant results (Table 3).

**Table 3:** Linear regression output. Response variable: DVP whole area; independent variable: endometrial thickness

	Estimate	Std. error	*t* value	Pr ( >|*t*|)
Intercept	34.811	1.8545	18.772	< 1.33e-14
Endometrial thickness	− 1.213	0.693	− 1.748	0.095

Residual standard error: 5.52 on 21 degrees of freedom

Multiple R-squared: 0.127, adjusted R-squared: 0.08

F-statistic: 3.057 on 1 and 21 DF, *p* value: 0.009

Bold values identify statistical significance

We found an interesting significant negative association between CC vessel density in the fovea area and insulin values (*p* = 0.0002) and glucose levels (*p* = 0.0335).

Furthermore, we found a negative association (statistically significant at 10% level) between DVP vessel density in the whole area and endometrial thickness values (*p* = 0.09).

## Discussion

Hypothalamic amenorrhea is responsible for a long and stable decrease in estrogen levels, leading to various consequences on the human apparatus as on the circulatory system regulation. In the eye district, two main vascular layers of the retinal plexus (superficial and deep) and choriocapillaris may be involved in vascular dysfunction.

The involvement of CC in FHA patients can be explained by the complexity of this district. Indeed, choriocapillaris is one of the most vascularized tissues in the human body [16]. Several changes in choriocapillaris vascular density have been described to be associated with other systemic diseases. Ramrattan et al. observed in their histological study that the density of the choriocapillaris decreased with aging [17].

In our study, we observed a statistically significant increased vessel density in CC both in the fovea and whole area in FHA patients compared to healthy controls, suggesting a direct correlation between CC vessel density and hypoestrogenism. A chronic condition of estrogen reduction induces a reduction of blood flow by vasoconstriction in many districts’ microcirculation. In the eye district, Kavroulaki and colleagues found choroidal blood flow significantly lower in menopausal women compared to women younger than 40 years [18]. On the other side, some evidences show that estrogen replacement therapy in postmenopausal women exerts salutary vasodilatory effects in different vascular districts as well as in the eye [19, 20]. It is possible that our findings of an increase in CC vessel density could be a compensation mechanism in face of the blood flow reduction at the choriocapillaris level with the aim of guaranteeing an adequate deep vascular support in such a critical retinal region.

Other significant results were derived by the correlation of the chorio-retinal vessel density data with laboratory findings. Particularly, a significant negative association between choriocapillaris vessel density and insulin values (*p* = 0.0002) and fasting glucose levels (*p* = 0.0335) for the fovea area was observed. Similarly to previous studies, we too found that insulin levels are often reduced in hypothalamic amenorrhea [21, 22]. Also, glucose levels are known to be lower in FHA patients [23]. Evidences that glycemic and insulin levels have an effect on eye microcirculation come from studies on diabetic patients and in particular from the diabetic retinopathy. Many authors have demonstrated a decreased vessel density in type II diabetic patients with no signs of diabetic retinopathy in both retinal and choroidal flow, suggesting an association between high glucose levels and eye microcirculation impairment [24–26]. Furthermore, Yang in 2022 found retinal vessel density evaluated by OCTA reduced after 3 months of aggressive insulin therapy in type II diabetic patients, suggesting an effect of high insulin level in blood on retinal vessel density [27].

We also found lower levels of prolactin in FHA patients (*p* = 0.001), although within normal limits. It is already known that patients affected by FHA may have lower levels of prolactine [5].

Interestingly, a difference in the deep vascular plexus whole area (at 10%, *p* = 0.096) was found between the two groups, with a negative association between DVP vessel density in the whole area and endometrial thickness values (at 10%, *p* = 0.095). The endometrial thickness can be considered as an indirect marker of estrogenization, and this negative association suggests that the increase in DVP is associated with hypoestrogenism. On the other hand, as already said, a regulatory role of estrogens on eye microcirculation has been demonstrated [16–18].

No other significant changes in SCP were observed in our group. This probably depends on the vascularization of this district, which is directly connected to the retinal arterioles, having a higher perfusion pressure and oxygen supply [27], or to a different autoregulation capacity which preserves this district from changes dangerous for the neuroretinal tissue.

It is important to notice that these vascular abnormalities were detected in the absence of ocular symptoms, so they can be considered pre-clinical alterations, and therefore can be used as a biomarker for any forthcoming clinically relevant alteration in eye vascularization.

Recovery of estrogen levels with estrogen replacement therapy could lead to a reversibility of these vascularization changes. In this perspective, future longitudinal interventional data are requested to investigate hormonal replacement therapy.

## Data Availability

Data are available on repositories, available on request.
